# Comparative transcriptomes of nine tissues for the Heilongjiang brown frog (*Rana amurensis*)

**DOI:** 10.1038/s41598-022-24631-6

**Published:** 2022-12-01

**Authors:** Wanyu Li, Yue Lan, Lei Wang, Lewei He, Ruixiang Tang, Megan Price, Bisong Yue, Zhenxin Fan

**Affiliations:** 1 Key Laboratory of Bioresources and Eco-Environment (Ministry of Education), College of Life Sciences, Sichuan University, Chengdu, 610064 Sichuan China; 2 Sichuan Key Laboratory of Conservation Biology On Endangered Wildlife, College of Life Sciences, Sichuan University, Chengdu, 610064 Sichuan China; 3 Sichuan Engineering Research Center for Medicinal Animals, Xichang, 615000 Sichuan China

**Keywords:** Computational biology and bioinformatics, Molecular biology, Zoology

## Abstract

The Heilongjiang brown frog (*Rana amurensis*) is widely used in traditional Chinese medicine. In particular, the oviduct and skin have been developed into various health products. However, limited numbers of complete genomes of amphibian species have been reported, excluding the Heilongjiang brown frog. Here, the transcriptomes of 45 samples from the liver, spleen, heart, ovaries, thigh muscles, skin, oviduct, stomach and intestine of five Heilongjiang brown frog were reassembled and analyzed. A total of 1,085,532 unigenes with an average length of 676.6 bp and N50 of 722 bp were obtained. Comparative transcriptomics of different tissues detected tissue-specific expression. There were 3248 differentially expressed genes (DEGs) in the ovary, and the number of unique DEGs between the ovary and spleen was the largest. The results of DEGs enrichment showed there were many pathways and items related to protein synthesis and metabolism in the oviduct. The DEGs of the skin were enriched with many bacterial defense items, indicating that there were a large number of antimicrobial peptides in the skin. Thus, these were suitable as biological sources for the development and extraction of antimicrobial peptides. Through the assembly of transcriptome sequencing data and functional annotation of the Heilongjiang brown frog genome, this study provides reference materials for further exploring and utilizing functional gene resources of frogs and lays a foundation for medical research and the development of new products.

## Introduction

Amphibian research has received more attention in the last few decades, and thus species descriptions have increase with roughly three new amphibian species being described per week^1^. As of 2020, approximately 8000 amphibian species have been described, but because their genomes are large and complex there are only ~ 13 published amphibian genomes and most assemblies are highly fragmented^2^. Only one genome is available from Ranidae (true frogs), the North American bullfrog (*Rana [Lithobates] catesbeiana*), which is surprising as it is the largest frog family with species found on every continent except Antarctica^3^. Sequenced frog genomes may be limited by the high cost of large-genome sequencing, yet transcriptome analysis is economically viable for studying the large and complex genomes of amphibians^4^. Indeed, transcriptome sequencing technology has been used in many amphibian species^5–7^, and has provided novel insights into their gene expression profiles and the evolution of their immune systems.

The Heilongjiang brown frog (*Rana amurensis*) belongs to Ranidae (Anura Amphibia) and is mainly distributed in Northeastern China. The frog is widely used in traditional Chinese medicine and is considered an invaluable economic animal with high medical value^8,9^. Its products have anti-fatigue, anti-lipid peroxidation, antitussive and expectorant effects on immune function and stress performance, and have been widely accepted by Chinese, and its comprehensive development prospect is broad as a traditional Chinese medicine^10^. Recent studies have focused on the frog’s oviduct, skin, kidney, muscle and other tissues, but mainly regarding nutritive material component analysis in these tissues^11–13^, scarcely any study about the genome sequence. Without its full genome sequence, the mechanism of action, gene expression mechanism and molecular regulation mechanism remain largely unknown. Here, we performed large-scale transcriptome sequencing of nine tissues (liver, spleen, heart, ovary, thigh muscles, skin, oviduct, stomach and intestine) from five Heilongjiang brown frogs using Illumina NovaSeq 6000 platform. We aimed to (1) perform de novo assembly to provide high quality transcriptome resources; (2) detect the gene expression and identify the differentially expressed transcripts between tissues; (3) investigate the pathways and functional terms related to tissue-specific characters. Our results exhibited tissue-specific gene expressions and provided a reference gene set with high quality, which would serve as a valuable resource for further genetic, genomic and medical research of the Heilongjiang brown frog and other frogs.

## Materials and methods

### Experimental design and sample collection

Five non-breeding adult wild female Heilongjiang brown frog were obtained from the foot of the mountain in Jiaohe, Jilin City, Jilin Province, China. The frogs were placed in a airtight container with CO_2_ for anesthesia and euthanasia. The liver, spleen, heart, ovaries, thigh muscles, skin, oviduct, stomach and intestine were dissected after anesthetized and euthanasia. The 45 samples from nine tissues were collected in cryotubes and immediately stored in liquid nitrogen for later RNA extraction. All animal experiments in this study were approved by the Ethics Committee of the College of Life Sciences, Sichuan University, number 20210309009. All experiments were performed in accordance with relevant guidelines and regulations. Our reporting of research involving animals follows the recommendations of the ARRIVE guidelines.

### Library preparation and RNA sequencing

Total RNA was isolated separately from the 45 samples using a TRIzol Kit (Invitrogen, CA, USA) according to the manufacturer's instructions. The RNA integrity was checked using an Agilent 2100 BioAnalyzer (Agilent Technologies, CA, USA), and the mRNA was purified from the total RNA using the PolyATract mRNA Isolation Systems Kit (Promega) following the manufacturer's instructions. Fragmentation was undertaken using divalent cations under elevated temperature in NEB Next First Strand Synthesis Reaction Buffer. First stand cDNA was synthesized using a random hexamer primer and M-MuLV reverse transcriptase (RNase H-). The second strand cDNA synthesis was then performed using DNA polymerase I and RNase H. In order to select cDNA fragments that were preferentially 150–200 bp in length, the library fragments were purified with AMPure XP system (Beckman Coulter, Beverly, USA). The adaptor modified fragments were selected by gel purification and amplified through PCR to create the final cDNA library. Transcriptome sequencing was conducted using Illumina NovaSeq 6000 according to the manufacturer's instructions.

### Sequence preprocessing and transcripts assembly

The 150-bp paired-end (PE) short reads were obtained after sequencing. We used NGS QC Toolkit v2.3.3^14^ with stringent criteria (reads with more than 90% bases within ≥ 20 base quality (Q-value)) to remove the low-quality paired-end reads or reads containing adaptors resulting in the clean reads. Samples of different tissues were combined, and de novo assembled with Trinity separately using the following parameters: -seqType fq -JM 200G -CPU 24 -min_contig_length 300, while the remaining were analyzed using default parameters^15^. The assembled transcripts of the nine tissues were clustered with CD-HIT-EST^16^, obtaining the final assembled unigenes. To evaluate the completeness, we employed Benchmarking Universal Single-Copy Orthologs (BUSCO; http://busco.ezlab.org/) to evaluate the gene set of the Heilongjiang brown frog using vertebrata data^17^. The protein coding regions prediction of assembled unigenes was carried out using TransDecoder-v5.5.0 and the longest open reading frame (ORF) predicted for each contig sequence with a minimum of 100 amino acids long^15^.

### Gene annotation

Unigenes from the de novo assembly were aligned to the NCBI (http://blast.ncbi.nlm.nih.gov/) non-redundant protein sequence (Nr) database^18^, Swiss-Prot (http://www.uniprot.org/)^19^ and Clusters of Orthologous Groups (COG; http://www.ncbi.nlm.nih.gov/COG/)^20^ by BLASTX with E-value cutoff of 1*10^−5^. Gene names were assigned to each protein sequence based on the best hit from all the alignment results. The resultant unigenes were searched against the Gene Ontology (GO; http://www.geneontology.org/) database^21^. To generate the Web Gene Ontology Annotation Plot (WEGO) software^22^ chart, the results from Blast2GO annotation were exported in WEGO native format. The WEGO chart was then generated by uploading to http://wego.genomics.org.cn/. Kyoto Encyclopaedia of Genes and Genomes (KEGG; http://www.genome.jp/kegg/pathway.html) databases^23^ annotation was performed using the single-directional best-hit (SBH) method in KEGG automatic annotation server (KAAS). KEGG Mapper* was used to reconstruct pathways.

### Differentially expressed gene and gene enrichment analysis

Processed reads from each sample were mapped to the assembled unigenes using HISAT2^24^. To obtain the expression raw read counts for each gene, expression abundance of genes was generated using Salmon^25^ with the SAM files created by HISAT2. In order to remove the calculation error, we removed the low expression genes from the final results, which met all the following conditions. (1) The number of samples with non-zero expression is less than 1/3 of the total number of samples. (2) The total expression raw read counts of the gene in all samples were less than 10. Based on the expression abundance of removing low expression genes, principal component analysis (PCA) from the plotPCA of DESeq2 R package^26^ was used to visualize the correlation of 45 RNA-seq samples. The DESeq2 R package was used to identify the different expression genes (DEGs) between the 9 different tissues, with raw read counts as the input. P values were corrected for multiple testing with the Benjamini–Hochberg false discovery rate (FDR ≤ 0.05) and an absolute value of log 2 fold change ≥ 2 was used to determine the significant differences in gene expression. GO and KEGG enrichment were performed by R package clusterProfiler^27^ with GO and KEGG annotation results of all genes of the Heilongjiang brown frog as the background gene set.

### Construction of co-expression network

Gene expression data after normalization by R of all 45 samples were used to construct multi-tissue co-expression networks that simultaneously captured intra- and inter-tissue gene–gene interactions. We ranked all genes according to MAD (median absolute deviation) value and selected the first 10,000 as representative genes^28^. These genes were clustered according to their dissimilarity and were divided into different modules. Modules with high similarity were merged and a topological overlap matrix (TOM) was constructed based on the merged modules. For each tissue, we calculated the connectivity of all genes in the modules with significant module − trait relationship (> 0.8) to identify hub genes. Cytoscape^29^ was then used to visualize the weighted correlation network for hub genes and find the key regulator (driver) genes. Meanwhile, GO and KEGG enrichment analysis of these hub genes were performed using the same method.

## Results

### Sequencing and de novo assembly

**Figure 1:**
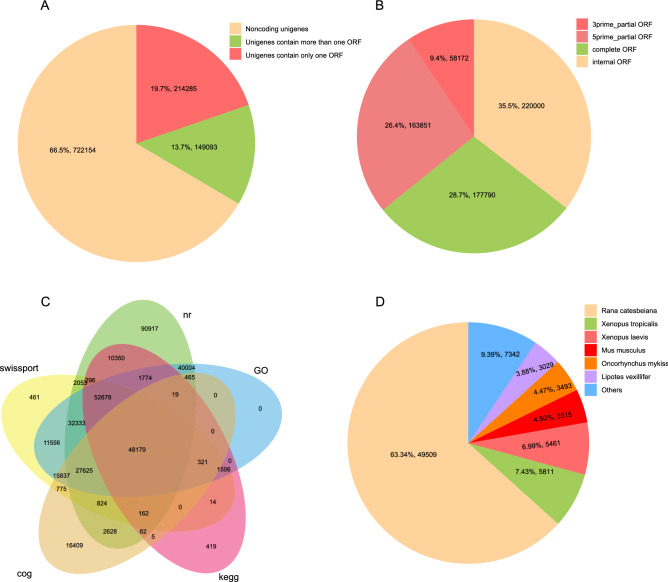
Coding gene prediction and annotation of unigenes. (**A**) Piechart showing the identification results of assembled unigenes coding ability by TransDecoder. (**B**) Piechart showing the classification of all identified open reading frames (ORFs). (**C**) Venn-diagram showing the overlap between results of unigenes aligned to NR, Swiss-Prot, KEGG, COGs and GO databases. (**D**) Species annotation results from unigenes aligned to NR database.

A total of 1,182,707,807 150 bp PE raw reads were generated. The sequencing depth is 6×. After quality-trimming and adaptor-clipping, 1,160,223,961 clean reads remained. The average Q20 and GC contents for the samples were 97.46% and 46.68% (respectively; Table S1). The assembled transcripts of the nine tissues were combined and clustered, and a total of 1,085,532 unigenes with an average length of 676.56 bp (N50 = 722 bp) and a total length of 734,428,057 nt were generated after splicing and removing redundancy (Table S2). BUSCO and homologous assessment showed that nearly 28.7% of total complete and single-copy BUSCOs were identified in this gene set and the fragmented BUSCOs and missing BUSCOs was nearly zero. However, the ratio of complete and duplicated BUSCOs was 68.5% (Fig. S1). The complete BUSCO is the sum of complete and single-copy BUSCO and complete and duplicated BUSCO (97.2%). A total of 1,239,626 ORFs were identified from 363,378 of the 1,085,532 assembled unigenes by TransDecoder, and 149,093 of the unigenes contained only one ORF (Fig. 1A, B).

### Functional annotation of unigenes

There were 357,673 unigenes obtained from the annotated samples based on sequence similarity. Among these, 310,369 (28.59%), 194,620 (17.93%), 115,785 (10.67%), 113,311 (10.44%) and 232,297 (21.34%) unigenes were aligned to NR, Swiss-Prot, KEGG, COGs and GO databases, respectively (Fig. 1C). The species with the highest similarity were obtained according to the NR annotation results (Fig. 1D), of which the top three species in Anura were: bullfrog (*Rana [Lithobates] catesbeiana*), tropical clawed frog (*Xenopus tropicalis*), African clawed frog (*Xenopus laevis*). Notably, the number of unigenes aligned to the bullfrog was greater than the sum of the other species.

The GO annotation showed that the major subcategories were “Cell (GO:0005623)”, “Cell part (GO:0044464)”, “binding (GO:0005488)”, “catalytic activity (GO:0003824)”, “cellular process (GO:0009987)” and “metabolic process (GO:0008152)” (Fig. S2). COG assignments were performed to predict and classify the possible gene functions (Fig. S3). Analysis of the COG annotations showed that there were 113,311 (10.44%) hits assigned by 1,085,532 unigenes. The hits from the COG prediction were classified into 26 functional categories, where the most enriched terms were general function (20,573 unigenes), followed by amino acid transport and metabolism (8,851 unigenes) and translation, ribosomal structure and biogenesis (8,829 unigenes).

### Identification of DEGs among tissues

RNA-seq data were first normalized to reduce the influence of technical noise, and then we performed a PCA on gene expression from the 45 samples. We found that the nine tissues clearly clustered by the first Principal Component (PC1) and PC2, which explained 48% of the variance (Fig. S4). To identify genes that displayed significantly different expression between different tissues, DEGs were analyzed with pairwise comparison of the nine tissues. A total of 82,903 significant DEGs were detected by DESeq2 (Table 1). DEGs with higher expression levels in one sample when compared to another were denoted as “upregulated”, while those with lower expression levels were termed as “downregulated”. In general, the number of DEGs (29,414) between ovary and spleen was the largest, while the number of DEGs (3917) between intestine and stomach was the smallest. The intersection of DEGs between one and the other eight tissues was defined as a unique DEG (UEG) of this tissue. We found that the stomach had the fewest UEGs (209) and the ovaries had the highest number (3248). We also detected 779 UEGs in the skin and 752 in the oviduct. Meanwhile these two tissues are key to the medicinal and edible value of Heilongjiang brown frog, and also illustrate the special characteristics of the skin and oviduct of Heilongjiang brown frog compared with other tissues at the molecular level, which will provide a foundation for further in-depth mining and development of Heilongjiang brown frog economic value and research on its medicinal mechanism.

**Table 1:** The numbers of differential expressed genes between samples.

	Intestine		Muscle		Liver		Ovary		Skin		Spleen		Oviduct		Stomach		Heart	
	Up	Down	Up	Down	Up	Down	Up	Down	Up	Down	Up	Down	Up	Down	Up	Down	Up	Down
Intestine	–	–	7856	12,014	4617	7867	7371	17,150	4963	6708	6274	9747	6396	9298	1750	2167	6383	9694
Muscle	12,014	7856	–	–	9600	8697	10,815	17,819	10,228	7852	10,299	8732	11,175	8716	9836	5930	4435	3670
Liver	7867	4617	8697	9600	–	–	9307	15,761	7032	5492	5295	6679	7842	7330	7316	4422	6576	6335
Ovary	17,150	7371	17,819	10,815	15,761	9307	–	–	17,573	7648	20,309	9105	17,456	8316	17,780	7436	18,581	8984
Skin	6708	4963	7852	10,228	5492	7032	7648	17,573	–	–	4331	6662	5634	6641	4690	3209	4836	6231
Spleen	9747	6274	8732	10,299	6679	5295	9105	20,309	6662	4331	–	–	9894	10,011	8606	4763	4429	3657
Oviduct	9298	6396	8716	11,175	7330	7842	8316	17,456	6641	5634	10,011	9894	–	–	6526	3148	8022	8415
Stomach	2167	1750	5930	9836	4422	7316	7436	17,780	3209	4690	4763	8606	3148	6526	–	–	4115	7414
Heart	9694	6383	3670	4435	6335	6576	8984	18,581	6231	4836	3657	4429	8415	8022	7414	4115	–	–
UEGs	537	21	1056	208	916	217	2003	1245	745	34	694	63	615	137	197	12	346	7
Hubgenes	37		154		125		273		21		64		86		19		49	

### GO category and KEEG pathway enrichment analyses

To gain insight into the biological roles of UEGs, we performed GO (Gene ontology) category and KEEG (Kyoto Encyclopedia of Genes and Genomes) pathway enrichment analyses.

The oviduct is one of the tissues we are most interested in. For the 752 UEGs of the oviduct, the upregulated genes of the oviduct were mainly enriched in regulation of endopeptidase activity (GO:0052548), microtubule polymerization (GO:0046785) and O-glycan processing (GO:0016266) (Table S18). The downregulated genes of the oviduct were mainly enriched in activation of adenylate cyclase activity (GO:0007190) and response to jasmonic acid (GO:0009753) (Table S19). Mucin type O-glycan biosynthesis (ko00512), glycosphingolipid biosynthesis—lacto and neolacto series (ko00601) and other glycan degradation (ko00511) were significantly enriched in the oviduct’s upregulated genes in KEGG enrichment (Table S18).

Likewise, we performed GO and KEGG enrichment with the 779 UEGs of the skin. The skin’s upregulated genes were mainly enriched in epidermis development (GO:0008544), antibacterial humoral response (GO:0019731) and defense response to gram-negative bacterium (GO:0050829) (Table S12). The skin’s downregulated genes were enriched in digestion (GO:0007586), taurine transport (GO:0015734) and modulation of chemical synaptic transmission (GO:0050804) (Table S13). Arachidonic acid metabolism (ko00590), drug metabolism—cytochrome P450 (ko00982) and isoquinoline alkaloid biosynthesis (ko00950) were significantly enriched in the skin’s upregulated genes highlighted in the KEGG enrichment analysis (Table S12).

Cardiac muscle contraction (GO:0060048), cardiac muscle tissue morphogenesis (GO:0055008) and regulation of heart rate (GO:0002027) were mainly enriched in the heart’s upregulated genes, found via GO enrichment analysis (Table S24). The UEGs of other tissues were also analyzed separately for GO and KEGG enrichment, refer to Tables S3–S29.

### Construction of gene co-expression modules and weighted correlation network analysis

**Figure 2:**
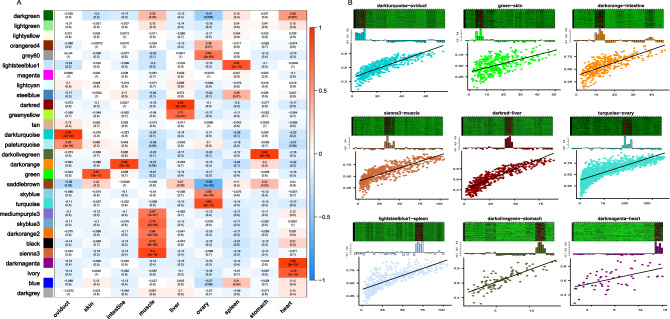
Analysis of gene co-expression modules. (**A**) Heatmap illuminates the association between modules and tissues. Each row corresponds to a module. Each column corresponds to a specific tissue. The color of each cell at the row-column intersection indicates the correlation coefficient between the module and the tissue. Red means significant correlation and blue means no significant correlation. (**B**) The internal situation of each module significantly related to the tissue (module − trait relationship > 0.8). The heatmap and histogram show the expression of all the genes belong to this module in each sample. The point graph visually shows the correlation between the module and tissue. (WGCNA_1.69: https://horvath.genetics.ucla.edu/html/CoexpressionNetwork/Rpackages/WGCNA/WGCNA_1.69-81.tar.gz).

We applied weighted gene co-expression network analysis (WGCNA) to gain further insight into the molecular mechanisms of each tissue^28^. After merging modules with high similarity, 10,000 selected genes were clustered into 29 co-expressed modules and the modules were denoted using different colors in Fig. S5. Each tissue showed significant module − trait relationship among the different traits (Fig. 2A). The relationships between module membership were shown in Fig. 2B, which also highlighted the connectivity of the most significant modules were correlated with tissues. For example, the turquoise module was strongly correlated with ovary tissue, which had the largest number of genes. DNA replication, meiosis—yeast and p53 signaling pathway were significantly enriched by the highly expressed genes (hub genes) in this module. The dark turquoise module was strongly correlated with oviduct tissue. Mucin type O-glycan biosynthesis and other glycan degradation were significantly enriched by the highly expressed genes in this module. (Table 1, Table S20).

**Figure 3:**
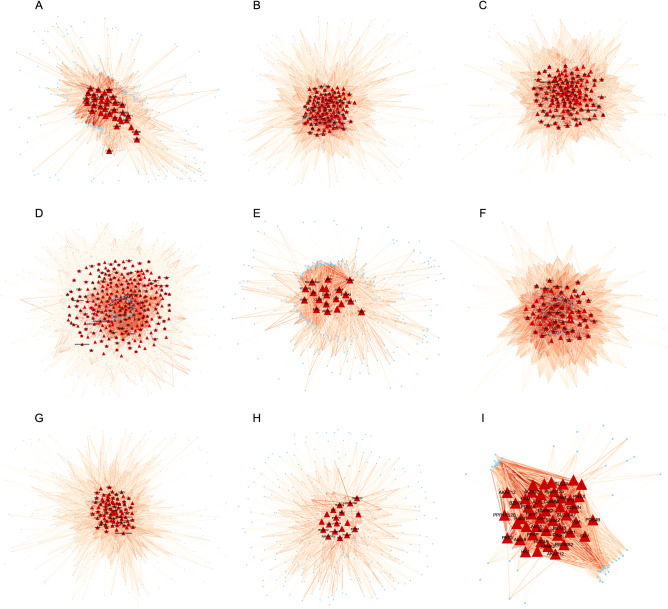
Weighted gene co-expression network of modules significantly correlate with tissues. The genes in network are not illuminated when the weight is less than 0.1. The triangle represents hub genes, which is statistically analyzed by WGCAN using cutoff of P value < 1e−8 and kME > 0.85 in each module. (**A**) Intestine. (**B**) Muscle. (**C**) Liver. (**D**) Ovary. (**E**) Skin. (**F**) Spleen. (**G**) Oviduct. (**H**) Stomach. (**I**) Heart.

Subsequently, hub genes in the modules that correlated with tissues were analyzed by co-expression network analysis (Fig. 3). The weighted correlation network showed that *CLDN1* (claudin 1) was the key gene of skin tissue, which functions as a major constituent of the tight junction complexes that regulate the permeability of epithelia^30^. *FUCA1* (Alpha-L-Fucosidase 1) was the key gene of oviduct tissue, which encodes a lysosomal enzyme involved in the degradation of fucose-containing glycoproteins and glycolipids^31^. *LMAN2L* (Lectin, Mannose Binding 2 Like) was the most important hub gene in the ovary, which functions in the mammalian early secretory pathway^32^. Within heart tissue, *CORIN* (Corin, Serine Peptidase) was in the most important position and is involved in the regulation of blood volume and pressure by encoding a member of the type II transmembrane serine protease class of the trypsin superfamily^33^.

## Discussion

RNA-seq analysis is an ideal high-throughput sequencing method, which provides high efficiency and rapidity for gene discovery and is useful for studying the large and complex genomes of amphibians^4^. In this study, RNA-seq technology was used to sequence the cDNA library of 45 samples from 9 tissues of five Heilongjiang brown frogs to construct a high-quality reference gene set and further analyze its tissue-specific expression. We obtained a relatively large transcriptome dataset, the large transcriptome may be the result of mixed splicing of different tissues. Additionally, it may be due to the complexity of the amphibian genome and more gene copies were produced with transposon element expansion and low loss rate^34^. In addition, our work also verified that RNA-seq can generate more new transcripts compared to traditional methods and more economical than genome sequencing, especially for species with large genomes^35^.

Comparison with NR, a non-redundant protein database, showed that a large number of annotated transcripts were aligned to proteins predicted by electronic annotation. This is due to the fact that the research on amphibians has not been in-depth yet, and the functions of a large number of proteins have not been verified by experiments. These annotation results are also inferred based on the similar functions of homologous proteins in different species. The matched number of unigenes of the bull frog was larger than the sum of all other species. The alignment results demonstrated the quality of the assembled unigenes and reflected the species similarity of relative species at the molecular level^36^.

Many differentially expressed genes in different tissues were also expected and reflected in the PCA analysis, the more DEGs between the two tissues, the farther they are in PCA plot. Because several tissues have similar functions (e.g. stomach and intestines, heart and thigh muscle) and thus their gene expression profiles are similar^37,38^. For example, after pairwise comparisons of nine tissues, we found that the smallest number of DEGs was between the intestine and stomach. However, some tissues are very different and thus we observed large differences in their gene expression profiles. For example, the ovary is a gonadal organ, which secretes estrogen and ovulates to produce oocytes^39^, while the spleen’s functions include digestion, absorption and transportation of food, filtration and storage of blood, and immunity^40^. Our results showed that there was almost no overlap in their enrichment results, and the number of DEGs between them was relatively large. The genetic divergence among the nine tissues of the Heilongjiang brown frog at the gene expression level would be helpful for the future research of its organ development.

Different tissues that were enriched in different GO terms and KEGG pathways were closely related to specific tissue functions and was consistent with similar studies in humans^41^.

The first example is the oviduct. We found that the UEGs in the oviduct were enriched in the extracellular domain, suggesting that the gene expression of the oviduct is more prone to exocytosis^42^. There was also a difference in the synthesis and secretion of several oviduct proteins compared to other tissues, which was consistent with a previous report^43^, which suggested the oviduct was a passive channel for sperm and egg transport and a highly active secretory organ. We also found that the most significant oviduct molecular function items were related to enzyme inhibitor activity and microtubule polymerization. Additionally, there were more protein synthesis activities in the oviduct compared to other tissues and that is why the oviduct is known for its strong protein secretion and metabolism^43^. In the biological process of GO enrichment, it was found that the oviduct had more glycosylation processes than other tissues. In particular, O-Glycosylation had an impact on a diversity of biological processes covering cellular aspects (targeted transport of glycoproteins), molecular aspects (protein conformation, resistance to proteolysis) and aspects involved in cellular communication (cell–cell and cell–matrix interaction) and was enriched by the upregulated genes of the oviduct. Cellular communication plays crucial roles in human infection by viral or bacterial pathogens (innate immunity) and in the progression of cancer^44^. The specific glycosylated protein may be related to the nutrition and medicinal function of the oviduct^45^. A large number of development-related items were enriched in the oviduct. These specific UEGs were probably the most fundamental reason for their special effects on activating cellular functions (treating scald, improving skin, anti-aging, etc.)^46^. In addition, some immune related GO terms were enriched, which could provide the support of the molecular regulation for the pharmacological mechanism of the oviduct. There were a small number of genes enriched in behavior and learning, and memory items, which indicates that the oviduct may have a special expression level in these aspects^47^. The two most significant pathways of KEGG annotation in the oviduct were glycosphingolipid biosynthesis globin series and O-type mucin glycan biosynthesis. Evidence for high expression of the mucin gene in the oviduct of *R. chensinensis*^48^ and *X. tropicalis*^49^ has been reported before. Mucin genes and O-type ligand glycosylation genes were likely candidates for maternal effects^50–52^. A variety of amino acid metabolic pathways, including related pathways of several essential amino acid (EAA), were found in the oviduct of the Heilongjiang brown frog. Therefore, it is likely that the oviduct has specific nutritional values and functions^53^.

Another example is the skin. We found that various enzyme inhibitors and regulatory activities, and a large number of items related to receptor binding were enriched in the skin, suggesting the tissue has a great capacity for protein synthesis. There were many receptors involved in protein expression regulation in skin, which were related to the metabolism, synthesis and secretion of proteins^54^. In the biological process of GO enrichment, the skin’s UEGs were mainly associated with bacterial defense response and external stimulus response. Previous study demonstrated that antimicrobial peptides were an essential part of the innate immune system in amphibians and were produced in response to a variety of external stimuli^55^. The rich stimulus response protein also further indicated that the skin of the Heilongjiang brown frog, as its own defense barrier, had a positive response to external stimuli. There were many immune defense regulatory mechanisms and expression products enriched in the skin. Jiang and Shang^56^ isolated Fraction III, a mixture of peptides from Chinese forest frog skin, and demonstrated that these bioactive peptides have antimicrobial activity against bacteria. Xiao et al.^57^ extracted five active peptide types from Chinese forest frog skin and found that the peptides had very high activity against gram-negative and gram-positive bacteria. These results indicate that the skin of amphibians is the main source of natural antimicrobial peptides and analogues. In the follow-up work, transcriptome data can be used to predict, screen and verify antimicrobial peptides from the skin of the Heilongjiang brown frog^58^. This research may highlight new varieties of antimicrobial peptides to support antibiotic development.

In conclusion, we assembled and annotated a fairly comprehensive gene set of the Heilongjiang brown frog. We also detected the gene expression differences among different tissues. The findings will provide basic data for genomics, gene cloning and functional verification of the Heilongjiang brown frog and other closely related amphibians.

## Supplementary Information

Supplementary Figure S1.

Supplementary Figure S2.

Supplementary Figure S3.

Supplementary Figure S4.

Supplementary Figure S5.

Supplementary Table S1.

Supplementary Table S2.

Supplementary Table S3.

Supplementary Table S4.

Supplementary Table S5.

Supplementary Table S6.

Supplementary Table S7.

Supplementary Table S8.

Supplementary Table S9.

Supplementary Table S10.

Supplementary Table S11.

Supplementary Table S12.

Supplementary Table S13.

Supplementary Table S14.

Supplementary Table S15.

Supplementary Table S16.

Supplementary Table S17.

Supplementary Table S18.

Supplementary Table S19.

Supplementary Table S20.

Supplementary Table S21.

Supplementary Table S22.

Supplementary Table S23.

Supplementary Table S24.

Supplementary Table S25.

Supplementary Table S26.

Supplementary Table S27.

Supplementary Table S28.

Supplementary Table S29.

## Data Availability

The raw RNA-seq data were deposited in the China National GeneBank DataBase (CDGBdb) with the accession number CNP0001589.
