# Supplementary figures and images for: Comparative transcriptomes of nine tissues for the Heilongjiang brown frog (Rana amurensis)
Source: Sci Rep. 2022 Dec 1;12:20759. doi: 10.1038/s41598-022-24631-6 (PMC9715712; doi:10.1038/s41598-022-24631-6)

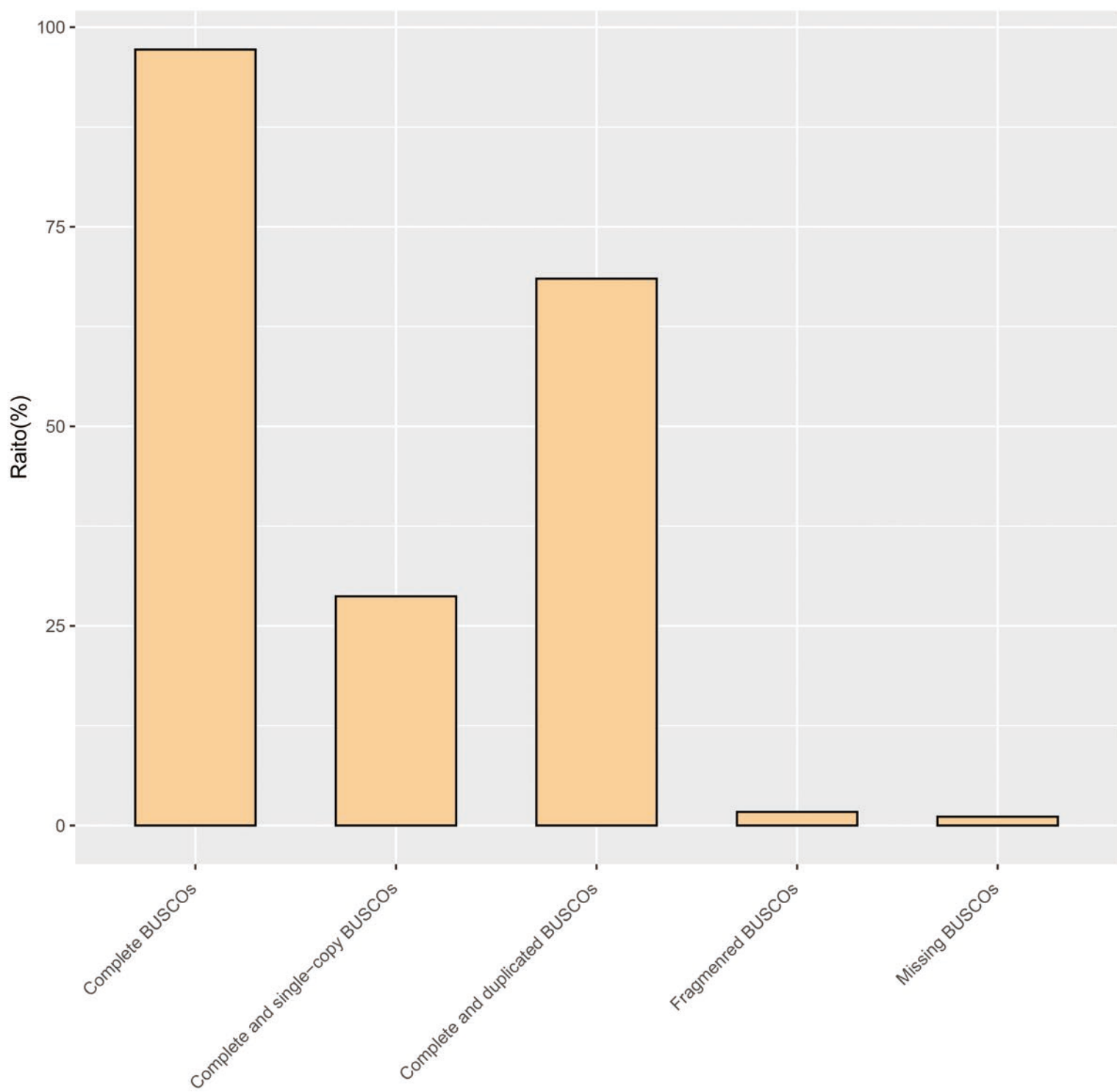

Figure S1. The results of BUSCO estimation.

Supplement: Supplementary file 1 — Supplementary Figure S1. [file 41598_2022_24631_MOESM1_ESM.pdf]

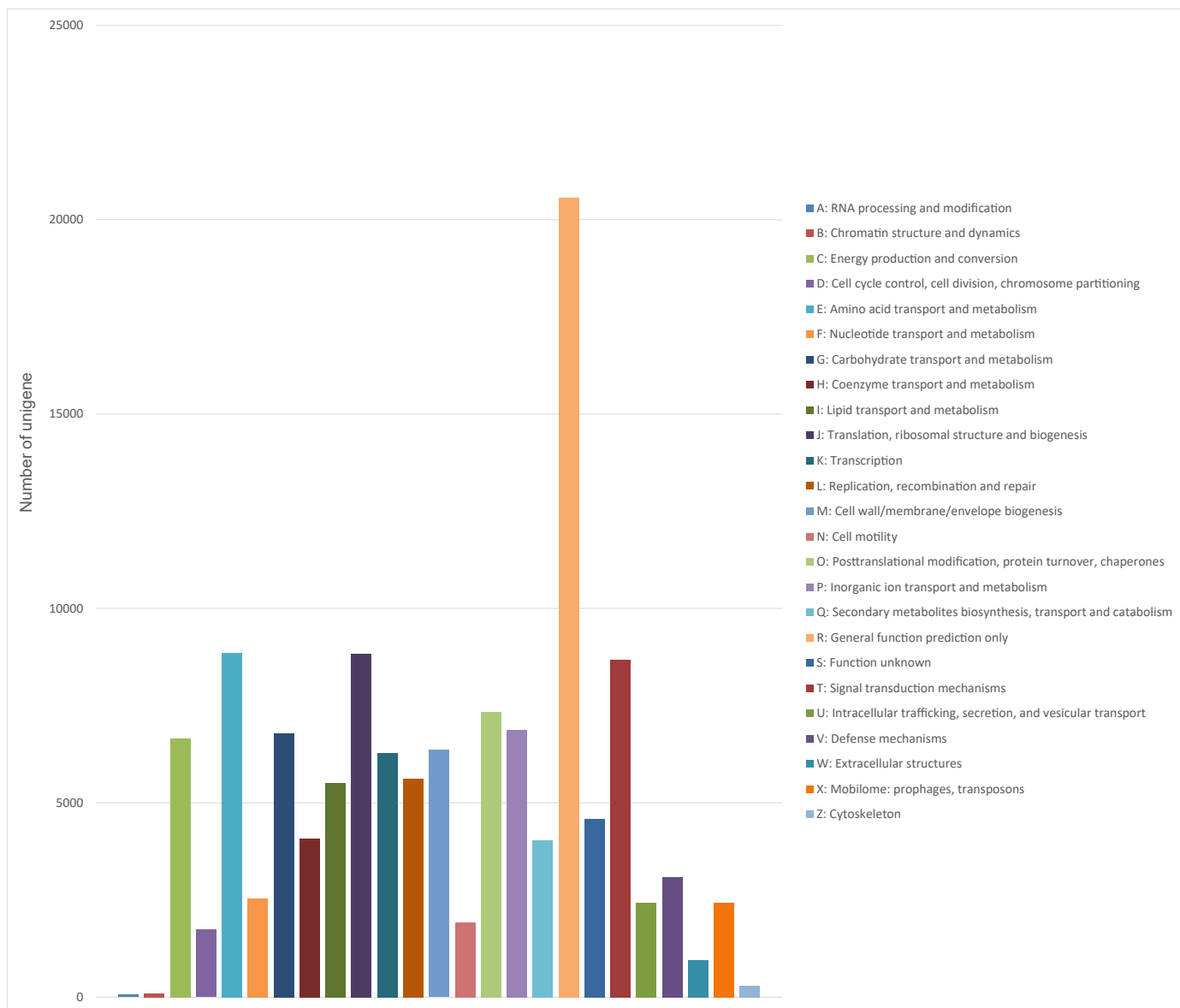

Figure S3. Bar chart showing the results of unigenes aligned to COGs.

Supplement: Supplementary file 3 — Supplementary Figure S3. [file 41598_2022_24631_MOESM3_ESM.pdf]

Cluster Dendrogram

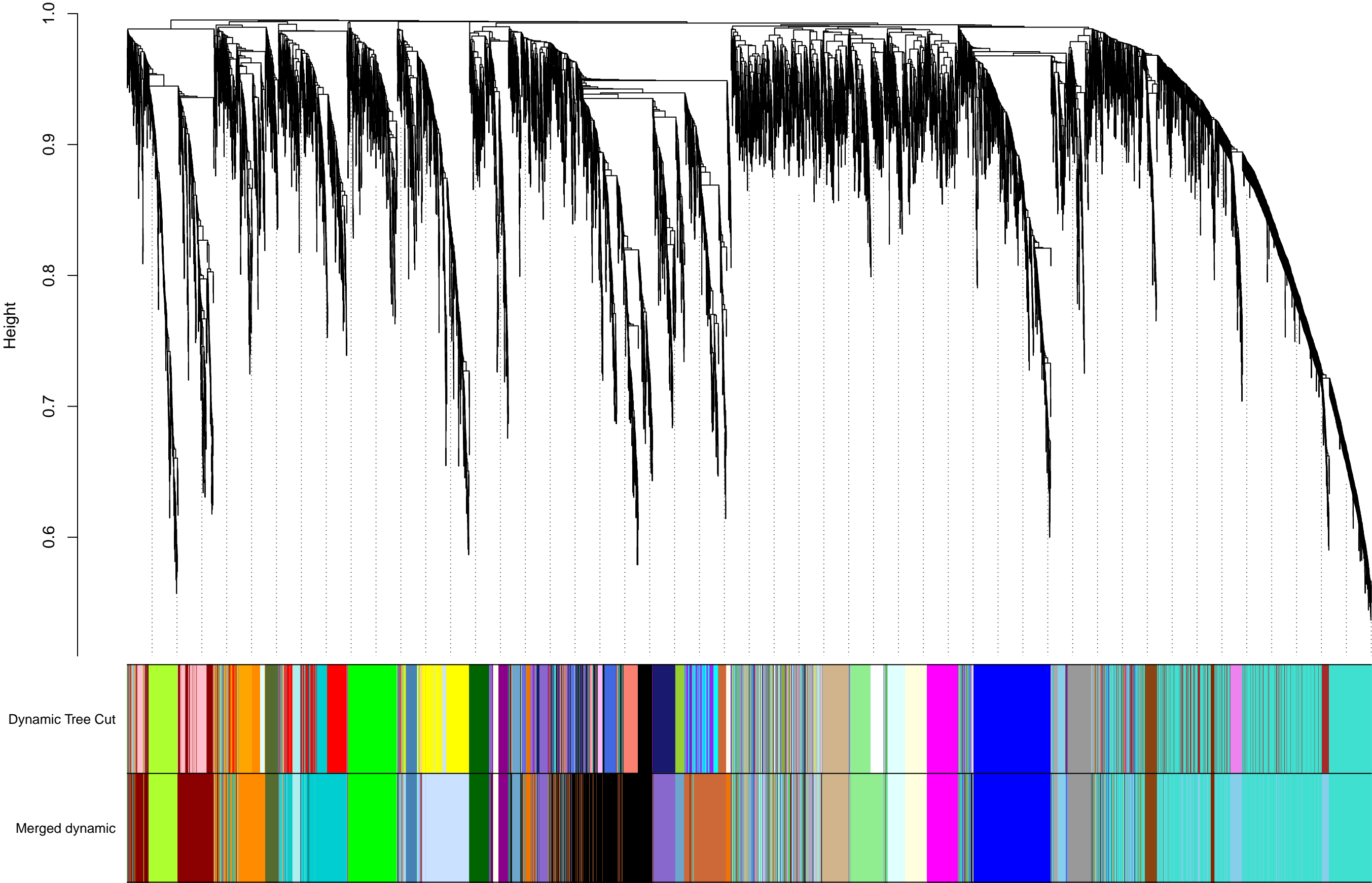

Figure S5. The result of gene clustering.

Supplement: Supplementary file 5 — Supplementary Figure S5. [file 41598_2022_24631_MOESM5_ESM.pdf]
