# Supplementary material for: Comparative transcriptomes of nine tissues for the Heilongjiang brown frog (Rana amurensis)
Source: Sci Rep. 2022 Dec 1;12:20759. doi: 10.1038/s41598-022-24631-6 (PMC9715712; doi:10.1038/s41598-022-24631-6)
Supplement: Supplementary file 2 — Supplementary Figure S2. [file 41598_2022_24631_MOESM2_ESM.pdf]

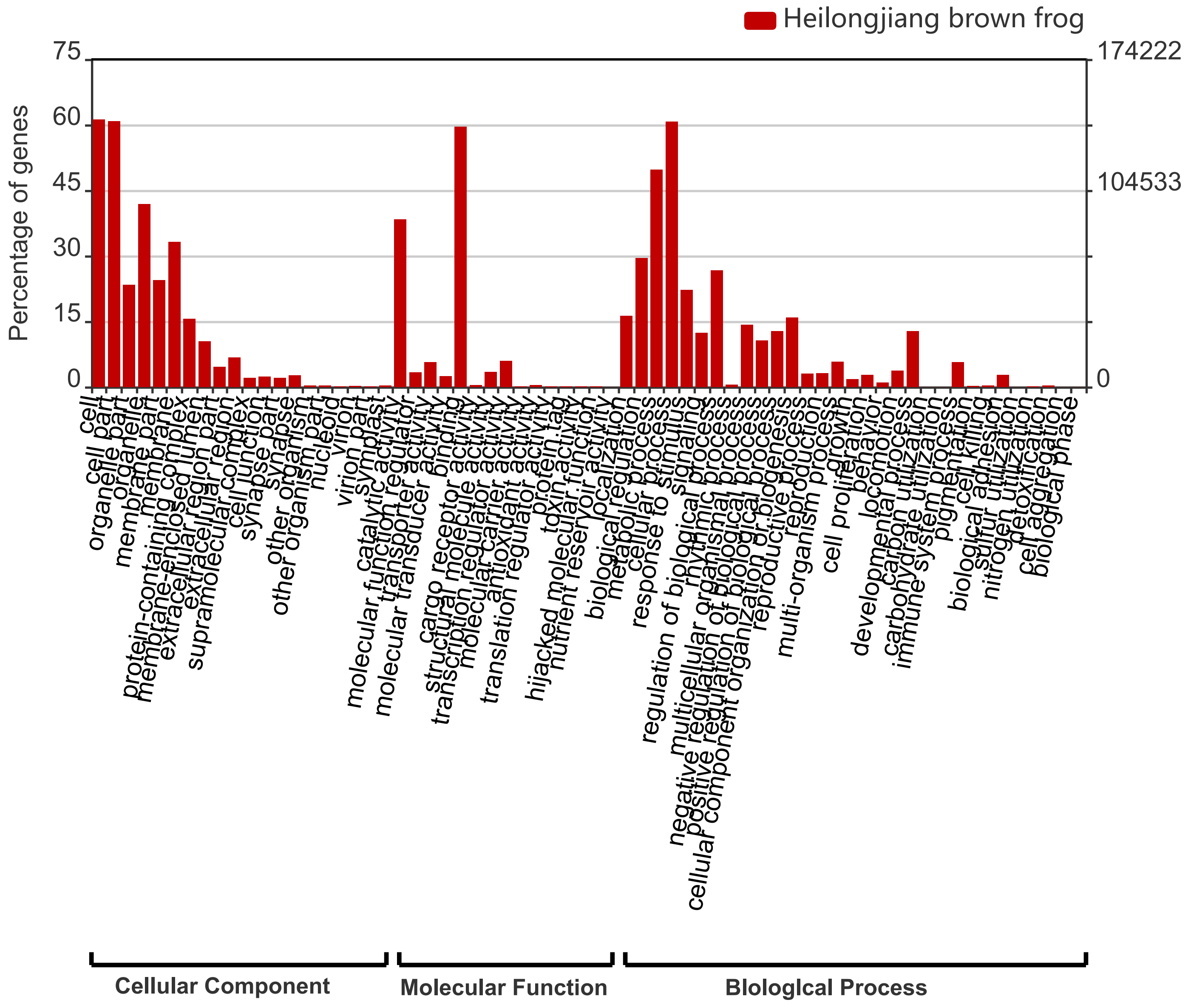

Figure S2. Functional classification of all the unigenes of the Heilongjiang brown frog based on Web Gene Ontology Annotation Plot.
