# Supplementary material for: Comparative transcriptomes of nine tissues for the Heilongjiang brown frog (Rana amurensis)
Source: Sci Rep. 2022 Dec 1;12:20759. doi: 10.1038/s41598-022-24631-6 (PMC9715712; doi:10.1038/s41598-022-24631-6)
Supplement: Supplementary file 4 — Supplementary Figure S4. [file 41598_2022_24631_MOESM4_ESM.pdf]

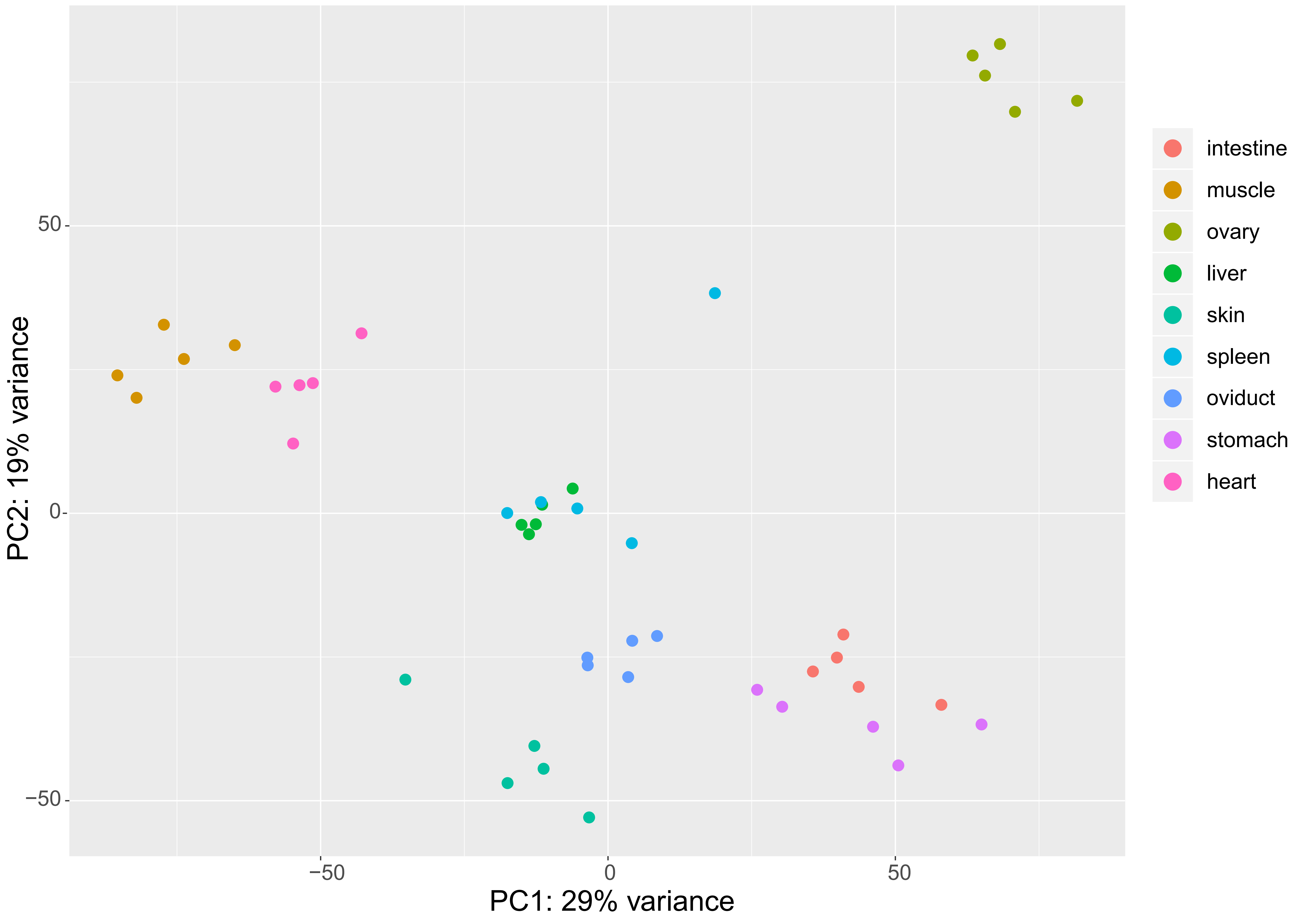

Figure S4. The principal component analysis (PCA) of 45 samples.
